# Therapeutics

**Published:** 1877-06

**Authors:** 


### II. Therapeutics.

*Atropia for Epilepsy*. Swetlin. (*Allg. Med. Centralztg*, 1877, No. 5).

In the clinic of Prof. Leidesdorf, a number of experiments made on Guinea pigs decidedly proved that atropia in small doses (one to four milligrammes) diminishes the action of the reflex nerve centres, while other experiments confirmed the well known fact that the long-continued use of larger doses of atropia (5 to 20 milligrammes) markedlv increases the reflex excitability. Some Guinea pigs made perfectly epileptic by Brown-Sequard's operation recovered completely in two or three weeks under the daily administration of 0,002 atropiae sulphas, while other Guinea pigs which were operated upon on the same day but were not medicated with atropia, continued to have the epileptic fits. Though the experiments with epileptic patients did not yield a complete success in every case, still the results were highly satisfactory in most cases. Recent cases of the purely motor epilepsy were always healed; in several cases of an inveterate epilepsy complicated by mental derangement, the recovery was complete, in others the number and severity of the attacks were greatly diminished. The remedy was prescribed as follows: Atrop. sulph. 0,05, pulv. et extr. gylcyrrh. q. s. ut f. pilul. 50; S. Take one pill daily.

The clinical observations show that the atropia given in daily doses of one milligramme may be continued for several months, without producing any signs of intoxication, and that this continued use of suchsmall doses is a very efficient treatment of epilepsy.

*Remedy for Headache.* J. E. Lockridge., M. D. (*American Practitioner*)

The doctor confines his remarks to idiopathic—so-called sick headache, or nervous headache, or neuralgic headache, and not the headache from organic trouble, nor to that accompanying any other disease, as fever, sore throat, small pox, etc, He says:

Many times have I been discouraged as well as worried with these cases. "Pain in my head," is the cry! Sometimes over the brow, sometimes through the temples or in the back of the head, or the whole head aches. Light and noise aggravate it; the room must be darkened and every one must walk noiselessly, suffering almost as much as the patient. Sometimes the head is hot; generally, I believe, there is no unnatural heat, except perhaps in the case of gentlemen who have spent a late evening over an extra glass of wine, or who have overtaxed their brains from a press of business. Sometimes there is nausea, attended or not with a slight coating on the tongue; just as often there is no nausea or other appreciable derangement of the digestive apparatus.

Now comes the perplexity and discouragement. We enjoin quietude and the exclusion of light and sound; we make cold or warm applications to the head as the case may be, and use the hot foot-bath and mustard-plasters to the nucha or temples; we give antacids, or indulge the patient with acids, as oranges, lemons, etc.; we try aromatic spirits of ammonia, lavender, valerianate of ammonia, compound spirits of ether, separately or combined; in short, we go through the whole list of the so-called nervines, antispasmodics and corrigents; but in spite of all, the headache pursues its own course in a vast majority of cases.

But now for my remedy. Having observed that bromide of potassium, in twenty or thirty grain doses, and tincture of aconite root, separately, relieved more cases than any remedies I had previously exhibited, I experimented with large doses of the drugs combined. For several years I have been in the habit of giving in these cases sixty grains of the bromide of potassium and ten drops of the tincture of aconite root, in a wineglassful of water; the same to be repeated in an hour or two, if the head be not relieved; but a repetition of the dose is very seldom required. In the case of ladies and others who wish to have the remedy always at hand, or who are about to start on a journey, I supply them with the following mixture:

℞ Bromide of potassium, . . . . ℥ ij

Tincture of aconite root, . . . ʒ j.

Distilled water, }

Simple syrup, } . . . . . āā ℥ ij.

M. S. Take a dessertspoonful in some water every hour, until relieved.

My recipe may smack of empiricism in appearing as a panacea for every variety of headache, let the cause be what it may and the accompanying symptoms be what they will; but I am willing for it to rest under the soft impeachment, if indeed it relieves promptly only a moiety of these distressing cases. I will not now attempt to give the *rationale* of this seeming paradox, or the *modus operandi* of the cure, but will simply remind my readers that this nervous headache is a paradoxical, capricious, discouraging and worrying affectlon.

*The Absorption of Mercurials. Dr. Hamburger, Franzensbad, Austria, (Prager Med. Wochenschrift.)*

The literature on the question whether mercury appears in the secretions during its use as a remedy, or not, is very extensive. The contradictory results that have been arrived at can be readily explained by the methods used in detecting mercury. Before electrolysis was used for this purpose, the reaction with sulphuretted hydrogen gas was the most sensitive test known. With this it was possible to detect 0.005 gr. Hg. Cl.$^2$ in 500 C. C. of distilled water, whilst the electrolysis 0.001 gr. Hg Cl.$^2$ in 500 be distinctly detected. Former experiments on this subject, with negative results, are only of value to us in that they show that mercury was not present in sufficiently large quantities to be detected with our present method of examination; however (electrolysis and afterwards changing the mercury to mercuric iodide), it must be remembered that only very small quantities need be present in order to make the objects to be examined, impure. Therefore all positive results must be taken with great hesitation. The author then goes on to state that the impurities may arise from the secretions, and also from the battery when that has amalgamated zinc elements. In order to avoid the latter contingency, it is necessary to keep the battery in a separate room from that in which the examination is made. The method used was that of Schneider. The substance to be examined was mixed with potassic chlorate and hydro-chloric acid, on the water bath, until all organic matter was destroyed; it was then evaporated until all the chlorine had escaped, water being constantly added, and then examined in the evaporating dish, with the electric current. When examining fæces only, the author filtered the solution. For electrolysis six Daniell cells were used to whose copper and zinc negative poles were attached, respectively platinum and gold wires. The current was allowed to act on the fluid for from 20 to 24 hours; after this time the gold wire was washed, placed between sheets of blotting paper and heated in a hermetically, sealed glass tube. After this gold wire was removed, iodine was introduced into the tube which was again sealed and heated. When mercury was present crystals formed could be recognized by means of the microscope without any difficulty. The results of the experiments are as follows:

*1st Experiment.* Franz Cz. . . . 24 years old. Has a papular syphilide, condylomata lata, a hard chancre and universal enlargement of the lymphatics; was treated with suppositories of 1.0 gr. mercurial ointment, which remained in the rectum from 20 to 24 hours. 6000 C. C. of urine were examined that were passed during the first four days of treatment. No mercury was found.

*2d Experiment.* After the same patient had been treated for two weeks with suppositories, the inunction cure was begun: 2.0 gr. of the ointment being used daily. The urine was collected from the eighth to the fifteenth day of treatment by this method. Mercury was present.

*3d Experiment.* Mrs. Anna Sch., 32 years old. Diagnosis: maculo-papular syphilide, universal enlargement of the lymphatics. The patient was then nursing her infant, aged four months. She was treated with suppositories. The urine examined was passed during the fifth to the fourteenth days of treatment. Mercury was detected.

*4th Experiment.* The milk of the same patient was examined and found to contain mercury.

*5th Experiment.* After fourteen days of treatment with suppositories, this method was given up, because the *eruption* was not disappearing, and recourse had to the method by inunction. After eight days of treatment by the latter method the urine was collected, and in it mercury found.

*6th Experiment.* During the same time, *i. e.*, between the eighth and eighteenth days, milk was taken from the patient. In it no mercury was found.

*7th Experiment.* J. H., 28 years old; has a hard chancre and a macular syphilide. The patient was put upon the inunction cure. The urine passed whilst using the first nine inunctions was collected, and mercury found in it.

*8th Experiment.* The fæces of the same patient were examined, and the presence of mercury distinctly demonstrated.

From these experiments the author draws the conclusions that, in order to affect the milk, suppositories ought to be used; that the mercury used by inunction never affects the milk, in which he supports the views of Dr. Kohler, but always is found in the urine and fæces. It seems that the excretion of mercury from the body takes place principally through the bile.—*Clinic.*

*Jaborandi in Bright's Disease.* Bruen. (*Philadelphia Med. Times,* April, 1877.)

Jaborandi as a remedy in Bright's disease, has found great favor with Doctor B., who reports seven cases successfully treated with the drug. He found prompt relief following the administration of an infusion of ʒii to ℥ii of water, the entire quantity given in one or two hours. About one hour after taking the tea, patient is usually bathed in perspiration, dyspnœa if any present, caused by fluid in the pleural cavity, is relieved, and the general condition of patient much improved. So thoroughly convinced is the author of the value of this remedy in dropsies, that he urges the profession to make use of it in private practice. W. F. L.

*Pulsatilla.* Wenzel. (*Louisville Med. News,* March, 1877.)

There are two preparations of this drug, the German tincture, and the American fluid extract. The tincture is chiefly used, and in ten-drop doses three times daily for several days, will produce the same results as small doses of hasheesh. In increased doses it causes frequent micturition and hematuria. Forty-drop doses of the tincture will cause violent headache, nervous excitement, and bloody stools. Severe headaches that have resisted all other remedies, will, the author says, receive benefit by from three to ten-drop doses of the tincture three times daily for one or two weeks. He believes it acts directly on the nerve centres, and principally on the cerebrum. Great care should be exercised in administering pulsatilla, because of its poisonous qualities. Doctor W. claims that wherever a nerve-sedative is required, no remedy is equal to it. W. F. L.

